# Low-Carbohydrate Diet Modulates Glucose–Lipid Utilization in Skeletal Muscle of Diabetic Mice

**DOI:** 10.3390/nu15061513

**Published:** 2023-03-21

**Authors:** Yan Li, Zi Yang, Yu Wang, Mingcong Fan, Chenzhipeng Nie, Lamei Xue, Li Wang, Haifeng Qian

**Affiliations:** State Key Laboratory of Food Science and Technology, School of Food Science and Technology, Jiangnan University, Wuxi 214122, China

**Keywords:** type 2 diabetes, low-carbohydrate diet, ketogenic diet, skeletal muscle, glucose utilization

## Abstract

Type 2 diabetes is associated with many complications, including skeletal muscle atrophy. Ketogenic diets and low-carbohydrate diets (LCD) have recently been introduced as dietary interventions in patients with diabetes, but their effects on glucose and lipid metabolism in skeletal muscle have not been studied. In the current study, we compared the effects of LCD and ketogenic diet on glucose and lipid metabolism in skeletal muscle of diabetic mice. C57BL/6J mice with type 2 diabetes, constructed by a high-fat diet combined with streptozotocin, were fed a standard diet, a high-fat diet, an LCD, or a ketogenic diet for 14 weeks, respectively. Here, we found that the LCD, rather than the ketogenic diet, retained skeletal muscle weight and suppressed the expression of atrophy-related genes in diabetic mice. In addition, the LCD had more glycolytic/type IIb myofiber content and inhibited forkhead box O1 and pyruvate dehydrogenase kinase 4 expression, leading to improved glucose utilization. However, the ketogenic diet maintained more oxidative/type I myofibers. Moreover, compared with the ketogenic diet, the LCD decreased intramuscular triglycerides content and muscle lipolysis, suggesting improvement in lipid metabolism. Taken together, these data suggested that the LCD improved glucose utilization, and inhibited lipolysis and atrophy in skeletal muscle of diabetic mice, while the ketogenic diet showed metabolic disorders in skeletal muscle.

## 1. Introduction

Type 2 diabetes is one of the chronic non-communicable diseases with the highest prevalence worldwide [1]. According to the International Diabetes Federation, the number of diabetic patients reached 463 million in 2019 (1 in 11 adults), and type 2 diabetes accounted for a large proportion among them. As one of the largest organs of the human body, skeletal muscle is in charge of up to 80% of postprandial glucose intake [2,3]. Impaired glucose uptake and utilization in skeletal muscle plays a vital role in the development of type 2 diabetes [4]. Skeletal muscle atrophy is one of the common complications of type 2 diabetes [5,6]. As the disease progresses, the lipid accumulation and chronic inflammation associated with type 2 diabetes further aggravate muscle atrophy [7,8]. Myofiber is the basic unit of skeletal muscle, according to the different subtypes of myosin heavy chain (*MyHC-I*, *MyHC-IIx*, *MyHC-IIa*, and *MyHC-IIb*), myofibers can be divided into four types, which include three fast-twitch fibers (type IIa, type IIb, and type IIx) and one slow-twitch fiber (type I). Different myofibers have different characteristics in metabolism. The slow-twitch fibers have strong oxidation ability due to its abundant mitochondria, while the fast-twitch fibers have higher glycolytic capacity [9]. Myofiber switch reflects specific metabolic states and affects the function of skeletal muscle. Some researchers found that with the development of type 2 diabetes, type I myofibers would transform into type II myofibers adaptively [10], but others believed that the improvement of glucose homeostasis was related to the increase of glycolytic myofibers [11,12]. Furthermore, the preferential loss and atrophy would occur in glycolytic, fast-twitch type II fibers during aging [13]. Thus, myofiber type transition is closely related to skeletal muscle metabolism and needs to be further studied.

People have been committed to taking dietary intervention as an adjuvant therapy for type 2 diabetes, and put forward a series of diet patterns, such as low-fat diet, calorie restricted diet, and so on. Dietary interventions have a profound impact on skeletal muscle metabolism. For example, Li Yinghui et al. demonstrated that a moderate reduction in dietary protein increased the expression of *MyHC-IIa* and *MyHC-I* in the skeletal muscle of the pig [14]. In addition, Li Yanjiao et al. found that the diet with low starch and high fiber could reduce the proportion of type IIb myofibers [15]. Recently, the ketogenic diet has come into our view. Different from traditional concepts, the ketogenic diet contains very low carbohydrate content and very high fat content, so that it could compel the body to use fat for energy and produce ketones. Many researchers have proposed that the ketogenic diet can reduce blood glucose in diabetic patients, but there are still many controversies concerning its effect on lipid metabolism and long-term applicability [16,17]. Thus, some others advocate a diet with a slightly lower fat content than the ketogenic diet (commonly known as the low-carbohydrate diet). They suggest that the low-carbohydrate diet (LCD) could also improve hyperglycemia and have better compliance [18]. The debate over these two diets has not yet been settled, and few studies have compared their influence on skeletal muscle metabolism in mice with diabetes. Therefore, it is urgent to explore the effects of ketogenic diet and LCD on myofiber conversion and glucose and lipid metabolism in skeletal muscle.

Skeletal muscle is responsible for both oxidative (mitochondrial) and non-oxidative (glycolytic) glucose disposal, which function is related to the ability to regulate fuel preference (metabolic flexibility) in the whole body [19,20]. Metabolic flexibility is often considered to be the ability to store and utilize fuel appropriately according to fuel availability and demand, which can enable the body to achieve the best substrate storage and utilization regardless of sufficient nutrition or lack of nutrition. Metabolic flexibility is vital for maintaining energy homeostasis; however, metabolic inflexibility is associated with many pathological states, such as obesity and type 2 diabetes [21]. Pyruvate dehydrogenase complex (PDHC) plays a key regulatory role in this process. It is one of the key rate-limiting enzymes in the glycolysis pathway, which catalyzes the irreversible oxidative decarboxylation of pyruvate to acetyl-CoA. PDHC activity decreases significantly in insulin resistance and type 2 diabetes, thereby inhibiting glucose utilization [22]. Furthermore, lipolysis defects and intramuscular lipid accumulation are both associated with type 2 diabetes, and may be part of the overall metabolic inflexibility [23]. Therefore, metabolic flexibility in type 2 diabetes requires more in-depth research.

In this study, we first constructed type 2 diabetic mice on a high-fat diet combined with streptozotocin (STZ), and then fed them four different diets. The results showed that the low-carbohydrate diet, rather than ketogenic diet, inhibited skeletal muscle atrophy. Moreover, the LCD had more type IIb fibers in skeletal muscle. Meanwhile, it promoted glucose utilization and suppressed lipolysis, but the ketogenic diet showed the opposite effects. In summary, our results indicated that the LCD and ketogenic diet exhibited different effects in myofiber type conversion and metabolic flexibility regulation, and the LCD may be more beneficial to the homeostasis of glucose and lipid metabolism in skeletal muscle.

## 2. Materials and Methods

### 2.1. Animals and Treatments

The male C57BL/6J mice used in the study were 4 weeks old and obtained from SLAC, Shanghai. All animal experiments were executed according to the protocols approved by the Laboratory Animal Ethics Committee of Jiangnan University (Approval Code: JN. No. 20190315c0320630 [24]). All mice were reared in a pathogen-free barrier facility, the temperature was between 20–24 °C and the relative humidity was between 45–55%. In the first two weeks, the mice had free access to water and chow diet.

To construct a mouse model of type 2 diabetes, the mice were fed a high-fat diet for 4 weeks to induce insulin resistance, then were intraperitoneally injected with STZ (Sigma–Aldrich, St. Louis, MO, USA) at a dose of 80 mg/kg. Seven days later, we selected some mice as diabetic mice and used in subsequent experiments, and the criteria is random blood glucose higher than 16.7 mmol/L [25].

The diabetic mice were randomly divided into 4 groups (n = 4/group): SD (standard diet), HF (high-fat diet), LCD (low-carbohydrate diet), and KD (ketogenic diet). All the groups were fed ad libitum for 14 weeks. The diets mentioned above were provided by TROPHIC (Jiangsu, Nantong, China), and the details of diet were in the supplemental file (Table S1).

Intraperitoneal insulin tolerance test (ITT) and intraperitoneal glucose tolerance test (GTT) were performed in vivo. After a four-hour fast or an overnight fast, venous blood of the tail was collected for detecting baseline glucose level at 0, 15, 30, 60, and 120 min after an intraperitoneal injection of glucose (1 g/kg body weight) or insulin (0.75 U/kg body weight). At the end of the experiment, serum samples and muscle tissues were collected and stored at −80 °C for subsequent determination.

### 2.2. RNA Isolation and qRT-PCR Analysis

Total RNA of tissues were extracted by Trizol reagent (Scientific Inc., Waltham, MA, USA) and its concentration was determined by a Nanodrop (Thermo Fisher Scientific). cDNAs were generated by PrimeScript RT reagent Kit (TaKaRa, Beijing, China) and quantified using SYBR^®^ Green Master Mix (TaKaRa, Beijing, China) and ABI 7900 system. All data were normalized to GAPDH expression. The primers were provided in supplemental file (Table S2).

### 2.3. Western Blotting

The total protein was extracted with RIPA (Beyotime, Shanghai, China) and the concentration was mesured by BCA (Beyotime, Shanghai, China). An equal amount of protein was resolved by SDS–PAGE and transferred onto PVDF membrane. Then, the membrane was incubated with the primary antibody and the second antibody respectively. As shown in Table S3, primary antibodies against Atrogin-1 (muscle atrophy F-box protein) and MuRF1(muscle RING finger-1) were purchased from Santa Cruz Biotechnology (CA, USA). Primary antibodies against GAPDH and PDK4 (pyruvate dehydrogenase kinase 4) were acquired from Proteintech (IL, USA). Antibodies against P-PDH, FoxO1 (forkhead box O1), PDH, ATGL (adipose triglyceride lipase), HSL (hormone-sensitive lipase), P-HSL, and HSP90 were purchased from Cell Signaling Technology (MA, USA). 

### 2.4. Measurement of Metabolic Profile

Fasting blood glucose levels of mice were measured under different feeding conditions. The levels of glycogen in skeletal muscle were measured based on the manufacturer’s instructions (Nanjing Jiancheng Bioengineering Institute, Nanjing, China). Skeletal muscle and serum levels of triglyceride were analyzed using commercially available kits.

### 2.5. Measurement of PDHC Enzyme Activity

PDHC activity was determined in accordance with the manufacturer’s protocols (ab109902, Abcam, Cambridge, UK). Briefly, tissue lysate samples were loaded on immunocaptured plate, incubated at room temperature for 3 h, and then the corresponding reaction solution was added. Then the absorbance at 450 nm was dynamically monitored.

### 2.6. Histological Analysis

Adenosine triphosphatase (ATPase) staining was used to determined muscle fiber types, and hematoxylin and eosin (H&E) staining was used to observe the pathological states of muscle.

### 2.7. Statistical Analysis

Results were expressed as mean ± SEM of at least three independent experiments. Student’s *t* test was performed to assess the difference between two groups. Data from more than two groups were analyzed by one-way analysis of variance (ANOVA) followed by Tukey’s test. *p* < 0.05 was considered statistically significant. GraphPad Prism 8.0 (GraphPad, San Diego, CA, USA) was used to all statistical analysis.

## 3. Results

### 3.1. Low-Carbohydrate Diet Inhibited Atrophy in Skeletal Muscle

**Figure 1:**
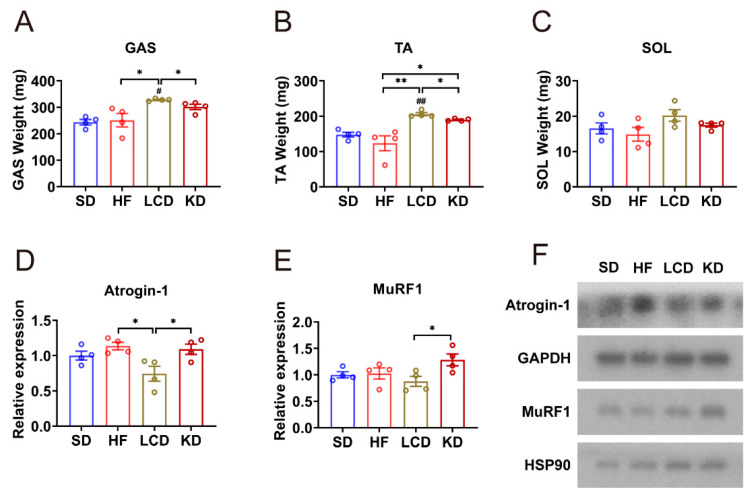
Low-carbohydrate diet inhibited atrophy in skeletal muscle. (**A**) GAS, (**B**) TA, and (**C**) SOL weight of mice with different treatments. (**D**,**E**) mRNA levels of (**D**) *Atrogin-1* and (**E**) *MuRF1* in the GAS muscle. (**F**) Western blots of Atrogin-1 and MuRF1 in the GAS muscle. Means ± SEM are shown. ^#^ *p* < 0.05, ^##^ *p* < 0.01 versus the standard diet group. * *p* < 0.05, ** *p* < 0.01 differences between groups. SD, standard diet; HF, high-fat diet; LCD, low-carbohydrate diet; KD, ketogenic diet.

After 14-weeks feeding under different conditions, we harvested the muscle tissue of the mice and weighed it at the same time. Much to our surprise, the weight of the GAS (gastrocnemius) muscle decreased significantly in the high-fat diet group and standard diet group. Moreover, compared with the LCD group, the GAS muscle weight in the ketogenic diet group was also remarkably reduced (Figure 1A). Similarly, by comparison with the LCD group, the weight of the TA (tibialis anterior) muscle in the standard diet group, high-fat diet group, and ketogenic diet group also declined to varying degrees (Figure 1B). As for the weight of the SOL (soleus) muscle, there was no significant difference among the four groups (Figure 1C). Therefore, we inferred that the low-carbohydrate diet inhibited atrophy in skeletal muscle. qPCR analysis was performed for further verification. *Atrogin-1* (muscle atrophy F-box protein) and *MuRF1* (muscle RING finger-1) are the key enzymes of muscle atrophy, and these two were significantly down-regulated in the LCD group (Figure 1D,E). In line with this, Western blot analysis exhibited the same results (Figure 1F). These results demonstrated that the LCD inhibited atrophy in skeletal muscle.

### 3.2. Low-Carbohydrate Diet Promoted the Percentage of Type IIb Fibers

**Figure 2:**
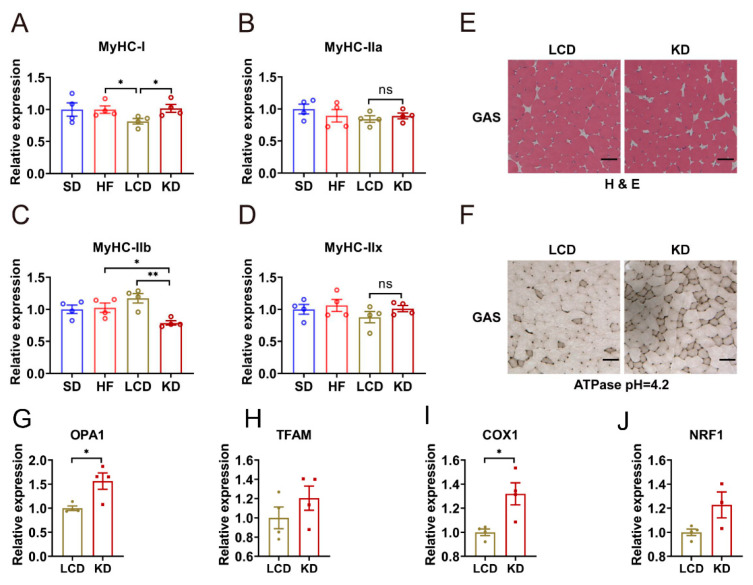
Ketogenic diet promoted the percentage of type I fibers. (**A**–**D**) mRNA levels of (**A**) *MyHC-I*, (**B**) *MyHC-IIa*, (**C**) *MyHC-Iib,* and (**D**) *MyHC-IIx* in the GAS muscle. (**E**) Representative images of H&E staining in the GAS muscle of the LCD group or ketogenic diet group. (**F**) Representative images of ATPase staining in the GAS muscle of the LCD group or ketogenic diet group. (**G**–**J**) mRNA levels of (**G**) *OPA1*, (**H**) *TFAM*, (**I**) *COX1,* and (**J**) *NRF1* in the GAS muscle. Means ± SEM are shown. * *p* < 0.05, ** *p* < 0.01 differences between groups, ns: no significance.

Skeletal muscle is composed of several different myofibers. Different myofibers have different metabolic characteristics. Under the influence of diet, nutrition, and other factors, myofiber types will change. We examined the influence of different diets on the type and distribution of myofibers. Figure 2A–D showed the results of qPCR analysis in skeletal muscle of mice with different treatments. Although there was no obvious change in the expressions of *MyHC-IIx* and *MyHC-IIa*, we could see that the LCD significantly reduced the expression of *MyHC-I* and up-regulated the expression of *MyHC-IIb* at the meantime. However, the ketogenic diet led to the opposite result. We also performed histochemical staining to observe muscle morphology. The results of H&E staining exhibited that the GAS myofiber in the LCD group were closely arranged, full in shape, and clear in structure (Figure 2E). However, in the ketogenic diet group, the cross-sectional area of myofibers decreased and the gap between myofibers became larger. ATPase staining was performed in an acidic environment, and it indicated that the amount of type I fibers in GAS muscle of the LCD group was significantly decreased compared with the ketogenic diet group (Figure 2F). Furthermore, type I fibers are abundant in in mitochondria and mainly rely on mitochondrial oxidative metabolism to produce ATP. The changes of mitochondrial markers with different diets were also detected. Consistent with previous results of qPCR, the ketogenic diet promoted the expression of genes related to mitochondrial number and activity compared with the LCD (Figure 2G–J). Consequently, these data suggested that the LCD increased the proportion of type IIb myofibers.

### 3.3. Low-Carbohydrate Diet Increased Glucose Utilization in Skeletal Muscle

**Figure 3:**
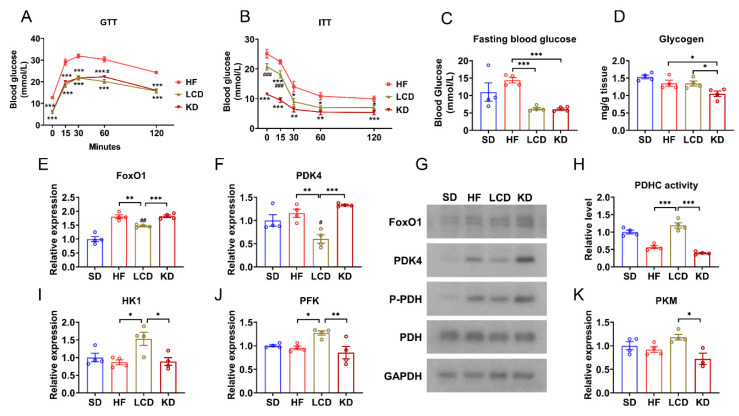
Ketogenic diet inhibited glucose utilization in skeletal muscle. (**A**) GTT of mice with different treatments. (**B**) ITT of mice with different treatments. (**C**) Fasting blood glucose of mice with different treatments. (**D**) Glycogen levels in the GAS muscle. (**E**,**F**) mRNA levels of (**E**) *FoxO1* and (**F**) *PDK4* in the GAS muscle. (**G**) Western blots of FoxO1, PDK4, and P-PDH in the GAS muscle. (**H**) PDHC activity in the GAS muscle. (**I**–**K**) mRNA levels of (**I**) *HK1*, (J) *PFK*, and (**K**) *PKM* in the GAS muscle. Means ± SEM are shown. In Figure 3A,B, * *p* < 0.05, ** *p* < 0.01, *** *p* < 0.001 versus the HF group. ^#^ *p* < 0.05, ^###^ *p* < 0.01 differences between LCD and KD groups. In Figure 3C–K, ^#^ *p* < 0.05, ^##^ *p* < 0.01 versus the standard diet group. * *p* < 0.05, ** *p* < 0.01, *** *p* < 0.001 differences between groups.

Because of the transition of myofiber type caused by different diet types, we further probed into the effects of diet on glucose metabolism in skeletal muscle. Compared with the high-fat diet group, the results of GTT and ITT have shown that LCD and the ketogenic diet actually improved glucose clearance or insulin response (Figure 3A,B), and the fasting blood glucose decreased significantly in the LCD group and the ketogenic diet group (Figure 3C). Equally notable was that the ketogenic diet evidently reduced glycogen levels in the GAS muscle (Figure 3D). We then determined the influence of different diets on *FoxO1*/*PDK4* metabolic pathway at mRNA and protein levels, which was involved in the process of fuel selection in skeletal muscle. Figure 3E and F showed that the LCD rather than the ketogenic diet decreased the expression levels of *FoxO1* and *PDK4* by qPCR analysis. This result was further confirmed by Western blot analysis and the expressions of FoxO1, PDK4, and P-PDH were down-regulated by the LCD (Figure 3G). Consistently, the PDHC activity of the LCD group increased compared with ketogenic diet (Figure 3H). Furthermore, the expression of glycolytic genes, hexokinase 1 (*HK1*), pyruvate kinaseisozyme M2 (*PKM*), and phosphofructokinase (*PFK*) was also investigated, and the results showed that the LCD increased the mRNA levels of HK1, PFK, and PKM (Figure 3I–K). The above showed that the LCD promoted the utilization of glucose in skeletal muscle.

### 3.4. Low-Carbohydrate Diet Decreased Lipolysis in Skeletal Muscle

**Figure 4:**
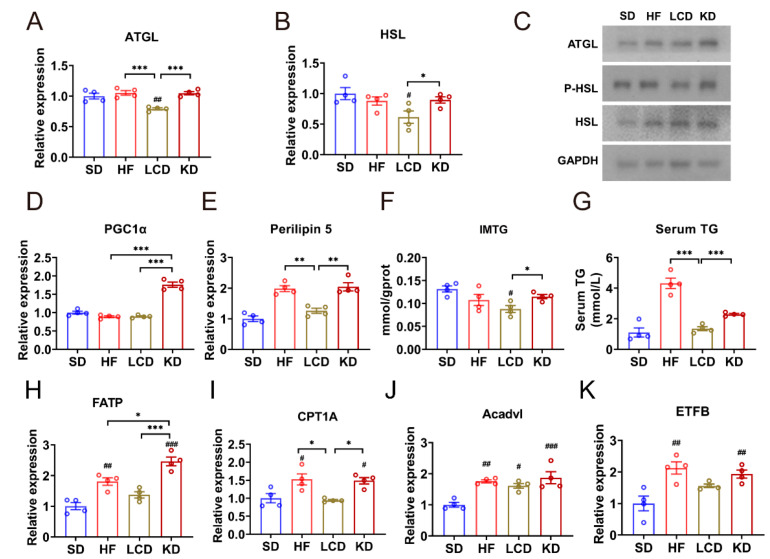
Ketogenic diet increased lipolysis in skeletal muscle. (**A**,**B**) mRNA levels of (**A**) *ATGL* and (**B**) *HSL* in the GAS muscle of mice. (**C**) Western blots of ATG, P-HSL, and HSL in the GAS muscle of mice. (**D**,**E**) mRNA levels of (**D**) *PGC1α* and (**E**) *Perilipin5* in the GAS muscle of mice. (**F**) Triglyceride content in the GAS muscle of mice. (**G**) Serum triglyceride content in mice of different treatments. (**H**–**K**) mRNA levels of (**H**) FATP, (**I**) CPT1A, (**J**) Acadvl, and (**K**) ETFB in the GAS muscle of mice. Means ± SEM are shown. ^#^ *p* < 0.05, ^##^ *p* < 0.01, ^###^ *p* < 0.001 versus the standard diet group. * *p* < 0.05, ** *p* < 0.01, *** *p* < 0.001 differences between groups.

Since the *FoxO1*/*PDK4*/*PDH* metabolic pathway plays an important role in metabolic flexibility, we went on to discuss the influence of different diets on lipid metabolism. Compared to the ketogenic diet, the LCD decreased the mRNA expression levels of *ATGL* (adipose triglyceride lipase) and *HSL* (hormone-sensitive lipase), which catalyze lipolysis (Figure 4A,B). Besides, the protein level also revealed a consistent downtrend in LCD; however, the protein expression of ATGL, P-HSL, and HSL in the ketogenic diet group was unbalanced (Figure 4C). In addition, reduced expression of *PGC1α* and perilipin 5 was also observed in the LCD group (Figure 4D,E). Figure 4F,G displayed the contents of intramuscular triglycerides and serum triglycerides, respectively. It was worth noting that the ketogenic diet did not reduce triglyceride content, but the LCD did. Furthermore, the mRNA expression of genes related to fatty acid oxidation in the GAS muscle of mice was also investigated and results exhibited that the ketogenic diet increased the mRNA expression of fatty acid transport protein (*FATP*), carnitine palmitoyl transferase 1A (*CPT1A*), acyl-coenzyme A dehydrogenase, very long chain (*Acadvl*), and electron transfer flavoprotein B (*ETFB*), indicating an increase in fatty acid oxidation (Figure 4H–K).

## 4. Discussion

Skeletal muscle is the most abundant tissue and plays a vital role in energy homeostasis in the body. It is the main site of insulin-mediated glucose treatment and fatty acid oxidation. Insulin resistance in muscle is one of the main hallmarks of type 2 diabetes, besides, abnormal skeletal muscle metabolism may affect the whole-body metabolism [26]. Therefore, we further investigated the influence of LCD and ketogenic diet on skeletal muscle. Muscle atrophy has been recognized as one of the major complications of diabetes [27]. Studies have shown that people with diabetes lose muscle mass twice as fast as those without diabetes [24]. Type 2 diabetes involves metabolic dysfunction, such as hyperglycemia and hyperlipidemia, which may lead to muscle atrophy [24,28]. In turn, muscle atrophy will further aggravate insulin resistance and diabetes. The interaction between muscle atrophy and diabetes forms a vicious circle. In our experiment, as in the high-fat diet group, the decreased GAS and TA muscle weight was observed in the ketogenic diet group, which was improved by the LCD group. Muscle atrophy is regulated by specific signaling pathways and transcriptional programs, mainly including two ubiquitin ligases, *atrogin-1* and *MuRF1* [29,30]. We measured the expression of these two enzymes to verify whether the LCD inhibited muscle atrophy. qPCR and Western analysis showed that LCD decreased the expression of atrophy related genes, while ketogenic diet increased the expression. In order to further observe the effects of LCD and ketogenic diet on the skeletal muscle of diabetic mice, H&E staining was performed on the skeletal muscle sections of these two groups of mice. The results showed that the GAS muscle fibers of mice in the LCD group were closely arranged, full in shape, and clear in structure, but the gap between GAS muscle fibers in ketogenic diet was larger than that in LCD, which supported the previous results. Therefore, we suggested that the LCD group inhibited skeletal muscle atrophy.

Skeletal muscle consumes energy mainly through the contraction of muscle fibers. Muscle fibers can be classified as type I and type II, which can switch to each other in different metabolic states. The relationship between diabetes and the transformation of muscle fiber type is still unclear. In fact, several studies have shown that a reduced percentage of type I fibers has been found in patients with diabetes. On the contrary, other studies have shown that increased glycolytic metabolism has the ability to improve systemic glucose homeostasis, and interventions to retain or restore glycolytic/fast myofibers may delay the metabolic diseases [31,32]. In this study, we analyzed the expression levels of four *MyHC* subtypes by PCR. We found that the LCD decreased the content of *MyHC-I* and increased the level of *MyHC-IIb*, while the ketogenic diet group changed in contrast, which meant the loss of glycolytic phenotype. The following ATPase staining further confirmed our results. ATPase staining is a common method to identify the types of skeletal myofibers. Type I myofibers rely mainly on aerobic metabolism for energy, so the activity of ATPase associated with contraction is low. Type II myofibers mainly rely on glycolysis for energy, so the activity of ATPase in muscle fibers is high. ATPase staining can stain type Ⅰ and type II myofibers into different shades of colors according to the activity of myosin ATPase. Under the condition of acid buffer (pH = 4.2), the color of type Ⅰ myofibers is darker than type II myofibers. Figure 2F shows the ATPase staining results of GAS muscle in mice fed with the LCD and the ketogenic diet. The proportion of type Ⅰ myofibers and mitochondrial biogenesis in the ketogenic diet group is significantly higher than that in the LCD group. This suggested that LCD and ketogenic diet had different effects on glucose homeostasis in skeletal muscle of diabetic mice.

qPCR and tissue section staining analysis showed that the ketogenic diet promoted the formation of slow-twitch fibers, while the LCD maintained more glycolytic fast-twitch fibers. The relationship between type conversion of skeletal myofibers and diabetes is still controversial. Some researchers have found that with the development of type 2 diabetes, type I myofibers will adaptively transform into type II myofibers, but others believe that the increase of type IIb myofibers is related to the improvement of glucose homeostasis. Therefore, we continued to explore which diet could improve glucose homeostasis of skeletal muscle in diabetic mice. 

Skeletal muscle is the place where most glycogen is stored in the body. Muscle glycogen is closely regulated by diet and exercise and has an impact on insulin sensitivity. The stored glycogen is an important energy source of skeletal muscle, which is broken down for energy when a lot of blood glucose is consumed during strenuous exercise. Figure 3B shows that there is no significant difference in muscle glycogen level between the LCD group and the high-fat diet group, while the muscle glycogen level in the ketogenic diet group is significantly lower than that in the high-fat diet group and the LCD group. The results of this paper are consistent with those of other studies on ketogenic diets. Kozue et al. found that in both short-term and long-term experiments, the ketogenic diet could significantly reduce the glycogen storage in skeletal muscle of mice [33]. S.D. Phinney et al. also found that short-term ketogenic diet therapy resulted in reduced skeletal muscle glycogen storage. In fact, these studies suggest that ketogenic diets reduce glycogen levels in skeletal muscle and inhibit anabolism of skeletal muscle [34]. In this study, we found that the LCD does not have these adverse effects and could maintain muscle glycogen content and skeletal muscle weight.

To further explore which diet could improve glucose homeostasis in diabetic mice, we investigated the effects of different groups on FoxO1/PDK4/P-PDH expression. *FoxO1* is associated with the conversion of muscle fibers and plays a vital role in glucose metabolism. *PDK4*, as a downstream target gene of *FoxO1*, is directly regulated by its transcription [35]. PDK4 is highly rich in skeletal muscle. Under hunger and some pathological conditions (such as insulin resistance and type 2 diabetes), PDK4 expression is significantly up-regulated. PDK4 could phosphorylate PDHC and then inhibit its activity, thus affecting the oxidative decomposition of glucose. The *FoxO1*/*PDK4* pathway inhibits glucose utilization in skeletal muscle by regulating PDHC activity [36,37], which eventually results in metabolic diseases [38]. Although both ketogenic diet and LCD reduced fasting blood glucose in diabetic mice, we suggested that the ketogenic diet increased FoxO1, PDK4, and P-PDH expression as did the high-fat diet, while LCD reduced the expression. 

The above shows that diet has an impact on PDHC phosphorylation level, so the PDHC activity in GAS muscle of diabetic mice treated with different diets was further measured. It can be seen from the figure that the PDHC activity of mice in the high-fat diet group is low, and in the LCD group, it is significantly higher than that in the high-fat diet group, while the PDHC activity in the ketogenic diet group has no significant difference with that in the high-fat diet group. PDHC plays roles in the regulation of glucose utilization in skeletal muscle. It links glycolysis to the tricarboxylic acid cycle, and catalyzes pyruvate, a product of glycolysis, to form acetyl-coA. The increase of PDHC activity could further up-regulate the glucose aerobic oxidation process. However, PDHC activity is significantly reduced in type 2 diabetes, leading to inhibition of glucose utilization. Thus, the ketogenic diet inhibited skeletal muscle glucose utilization, while the LCD promoted this process. Overall, our results suggested that LCD retained more glycolytic/type IIb myofibers and promoted glucose utilization.

The abnormal lipid content in skeletal muscle is related to insulin resistance and the occurrence of type 2 diabetes, and the intramuscular triglyceride content can better predict the insulin resistance of skeletal muscle than the fat content. In skeletal muscle, the intermediate product of lipid metabolism will weaken the insulin signaling of skeletal muscle, thus blocking the glucose uptake, and leading to the disorder of glucose and lipid utilization of skeletal muscle. Our experiment showed that LCD inhibited the accumulation of intramuscular triglyceride. Under normal circumstances, in response to insulin stimulation, skeletal muscle will shift from lipid catabolism to increased glucose oxidation and storage. However, diabetic patients will not be able to complete this transition and exhibit increased lipolysis [39]. In addition, the imbalance of the expression of ATGL and HSL will further deteriorate insulin resistance. In this study, their expression decreased in skeletal muscle of mice fed with LCD. This may be related to the increased ability of insulin to inhibit lipolysis [40]. mRNA levels of *PGC1α* and Perilipin 5 further confirmed the decrease of lipolysis. The ketogenic diet, however, led to unbalanced expression of ATGL and HSL at protein level. This further indicated that ketogenic diet led to metabolic disorder of skeletal muscle.

The data mentioned above suggested that the ketogenic diet shifted metabolism toward more lipid oxidation. Of course, the glycolysis–oxidation metabolic transition is an adaptive response to certain conditions in some cases, such as fasting. Phelix et al. also proved that endurance training athletes could increase lipid oxidation due to lipid overload, but they retained glycogen storage in muscle [41]. However, the ketogenic diet decreased the glycogen content in GAS muscle, which may indicate the destruction of glucose homeostasis and metabolic flexibility.

In conclusion, our data suggested that the ketogenic diet forced metabolism to change from glycolysis to lipid oxidation, which may lead to disorders in glucose and lipid metabolism. However, the LCD maintained more glycolytic/type IIb myofibers and improved glucose utilization. In addition, the LCD improved diabetes-related skeletal muscle atrophy. Therefore, our study provided a new view for dietary intervention in type 2 diabetes. Since type 2 diabetes is also characterized by insulin resistance, the changes of key factors of insulin signaling with different diets should be further studied. 

## Data Availability

The data that support the findings of this study are available from the corresponding author upon reasonable request.

## Supplementary Materials

The following supporting information can be downloaded at: https://www.mdpi.com/article/10.3390/nu15061513/s1, Figure S1: Raw images of western blotting; Table S1: Diet compositions; Table S2: Primer sequence; Table S3: Primary antibodies.
