# Supplementary material for: Low-Carbohydrate Diet Modulates Glucose–Lipid Utilization in Skeletal Muscle of Diabetic Mice
Source: Nutrients. 2023 Mar 21;15(6):1513. doi: 10.3390/nu15061513 (PMC10051166; doi:10.3390/nu15061513)
Supplement: Supplementary file 1 [file nutrients-15-01513-s001.zip › Supplementary material.pdf]

Table S1. Diet compositions.

Compositions	SD	HF	LCD	KD
Casein (g)	137	268	156	176
Methionine (g)	3	4	5	5
Corn starch (g)	569	36	14	0
Dextrin (g)	95	134	92	0
Sucrose (g)	63	90	102	2
Fat (g)	40	337	480	645
Cellulose (g)	47	67	77	87
Minerals, vitamin (choline) (g)	46	64	75	85
Tert-butylhydroquinone (g)	0.01	0.07	0.1	0.13
Total (g)	1000	1000	1000	1000

Table S2. Primer sequence.

Gene	Sense Sequence (5'→3')	Antisense Sequence (5'→3')
Atrogin-1	GAGTGGCATCGCCCAAAAGA	TCTGGAGAAGTTCCCGTATAAGT
MuRF1	TGGAGGTCGTTTCCGTTGC	CTGAGGTTCTGTCTGCGGTAG
FoxO1	TCAAGGATAAGGGCGACAGC	TGTCCATGGACGCAGCTCTT
PGC1α	CAATGAATGCAGCGGTCTTA	GTGTGAGGAGGGTCATCGTT
MyHC-I	ACTGTCAACACTAAGAGGGTCA	TTGGATGATTTGATCTTCCAGGG
MyHC-IIa	AAGTGACTGTGAAAACAGAAGCA	GCAGCCATTTGTAAGGGTTGAC
MyHC-IIb	TTGAAAAGACGAAGCAGCGAC	AGAGAGCGGGACTCCTTCTG
MyHC-IIx	CTCCAGGCTGCTTTAGAGGAA	CCTGCTCCTAATCTCAGCATCC
PDK4	AGGGAGGTCGAGCTGTTCTC	GGAGTGTTCACTAAGCGGTCA
ATGL	GTGTCAGACGGCGAGAATG	TGGAGGGAGGGAGGGATG
HSL	TCCCTCAGTATCTAGGCCAGA	GGCTCATTTGGGAGACTTTGTTT
Perilipin 5	GTGACTACCTGTGCCCTGGAC	TGATGGCTGCTGCAGGAAG
TFAM	GAGCAGCTAACTCCAAGTCAG	GAGCCGAATCATCCTTTGCCT
NRF1	AGCACGGAGTGACCCAAAC	AGGATGTCCGAGTCATCATAAGA
COX1	CAGAAGGCGCTGAAGGAGAA	ATCAGAACGAGCGCAGTGAA
OPA1	TGGAAAATGGTTCGAGAGTCAG	CATTCCGTCTCTAGGTTAAAGCG
HK1	CGGAATGGGGAGCCTTTGG	GCCTTCCTTATCCGTTTCAATGG
PFK	TGTGGTCCGAGTTGGTATCTT	GCACTTCCAATCACTGTGCC
PKM	GCCGCCTGGACA TTGACTC	CCATGAGAGAAATTCAGCCGAG
FATP	TATATGCTGGACCTTCGCACA	CTCCCCGCCATAAATGAGGG
CPT1A	AGATCAATCGGACCCTAGACAC	CAGCGAGTAGCGCATAGTCA
Acadvl	ACTACTGTGCTTCAGGGACAA	GCAAAGGACTTCGATTCTGCC
ETFB	TGTGGTGACTGCTGACCTA	TGTGGTGACTGCTGACCTA

Table S3. Primary antibodies.

Protein	Supplier	catalogue no.	dilution
Atrogin-1	Santa Cruz Biotechnology	sc-166806	1: 1000
GAPDH	Proteintech	10494-1-AP	1: 5000
MuRF1	Santa Cruz Biotechnology	sc-398608	1: 1000
PDK4	Proteintech	12949-1-AP	1: 1000
P-PDH	Cell Signaling Technology	31866	1: 1000
FoxO1	Cell Signaling Technology	2880	1: 1000
PDH	Cell Signaling Technology	2784	1: 1000
ATGL	Cell Signaling Technology	2439	1: 1000
HSL	Cell Signaling Technology	18381	1: 1000
P-HSL	Cell Signaling Technology	4139	1:1000
HSP90	Cell Signaling Technology	4874	1: 1000

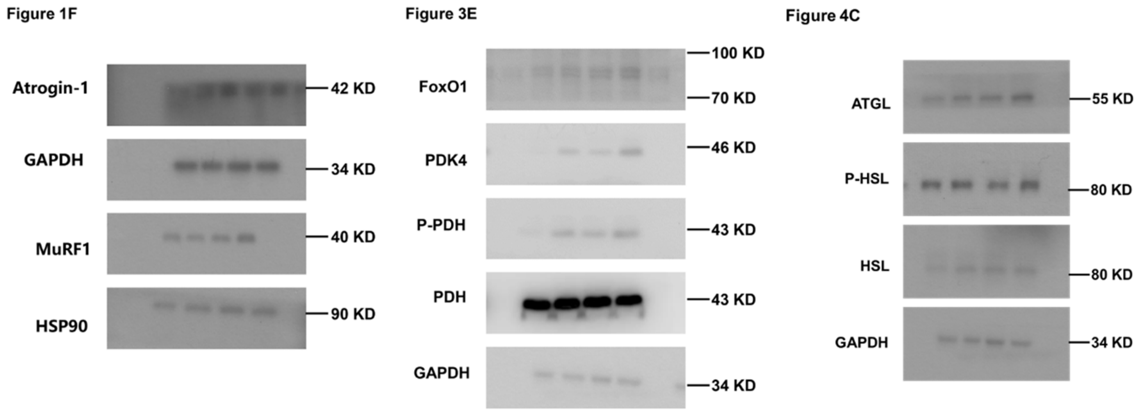

Figure S1. Raw images of western blotting.
